# Sealing efficacy of a single-cone root filling after post space preparation

**DOI:** 10.1007/s00784-015-1593-2

**Published:** 2015-09-28

**Authors:** D. Deniz Sungur, A.-T. Moinzadeh, P. R. Wesselink, S. Çalt Tarhan, A.R. Özok

**Affiliations:** Department of Endodontics, Faculty of Dentistry, Hacettepe University, Hacettepe Üniversitesi Diş Hekimliği Fakültesi Endodonti AD. 4. Kat, Sıhhiye, 06100 Ankara, Turkey; Department of Endodontology, Academic Centre for Dentistry Amsterdam (ACTA), University of Amsterdam and VU University Amsterdam, Gustav Mahlerlaan 3004, 1081 LA Amsterdam, The Netherlands

**Keywords:** Fluid transport, Root canal sealer, Root post space, Sealing efficacy, Single-cone, Wetting fluid

## Abstract

**Objectives:**

The aim of the present study was to compare the sealing efficacy of root fillings made by a single-cone technique with three different sealers and a cold lateral compaction technique with an epoxy sealer.

**Materials and methods:**

Eighty extracted single-rooted human teeth were assigned to four experimental groups: group 1, single-cone and epoxy sealer; group 2, single-cone and calcium silicate-based sealer; group 3, single-cone and methacrylate resin-based sealer; and group 4, cold lateral compaction and epoxy sealer. Twenty extra teeth served as negative and positive controls. After preparation of a coronal post space, the sealing efficacy of the root fillings was assessed on a fluid transport setup. The results were analyzed using Kruskal-Wallis and Mann-Whitney *U* test.

**Results:**

No fluid transport was detected for the negative controls whereas all the positive controls showed rapid fluid transport. No significant difference was detected between groups 1, 3, and 4 whereas group 2 demonstrated significantly more fluid transport than all the other experimental groups.

**Conclusions:**

Root fillings made by a single-cone technique with the epoxy or methacrylate-based sealers were as effective after post space preparation as those made by a cold lateral technique with the epoxy sealer in sealing the root canal.

**Clinical Relevance:**

Specific root canal sealers in combination with single-cone technique represent a noteworthy alternative to the use of cold lateral compaction technique when a post space is required. The use of effective endodontic procedures with simplified technical implementation may positively affect endodontic outcome.

## Introduction

Endodontically treated teeth may require the placement of a post and core for restorative purposes [1]. Preparation of a post space necessitates the removal of the coronal part of the root canal filling. The remaining apical root filling material then solely provides the seal between the post-build-up complex and the periapical tissues. Since shorter sections of root filling may represent a weaker barrier to coronal leakage, it is essential to use materials and techniques that can provide an adequate seal of the apical root canal third when a post is required.

The cold lateral compaction technique is often used as a gold standard to which other techniques are compared. This technique provides a relatively good adaptation of the filling material to the dentinal wall in the root canal apical third [2]. It remains, however, operator-dependent, and the forces resulting from the compaction component may provoke damage to root canal dentin [3]. A simple and most probably less damaging alternative to this technique is the use of a gutta-percha cone of greater taper with dimensions matching to those of the last instrument used during root canal preparation. This modus operandi is commonly named the single-cone technique and does not involve any compaction component. It can, therefore, be considered as a method that is less operator-dependent and potentially less damaging to the dentinal wall. When a single-cone technique is used, one relies on a sealer with adequate physical and chemical properties, to flow and fill the interfaces between the cone and dentin in order to provide a tight seal. Several commercially available sealers with different adhesive mechanisms have been designed to be used in this fashion. EndoREZ (Ultradent Products, South Jordan, UT, USA) is a dual-cure urethane dimethacrylate (UDMA)-based sealer. The close contact between UDMA molecules and the collagen fibrils of dentin is partly responsible for its adhesive properties [4]. AH26 (Dentsply DeTrey, Konstanz, Germany) is an epoxy amine resin-based sealer. The creation of covalent bonds between epoxy molecules and the amino groups of collagen may partly explain the relatively strong adhesion values observed with this sealer [5]. Another type of adhesion has been described with calcium silicate-based sealers such as EndoSequence BC Sealer (Brasseler USA, Savannah, GA, USA). These are expected, by interacting with dentinal fluids, to create and deposit intrafibrillar apatite and form tag-like structures within dentin, characterizing thus their bioactivity [6]. Improvements in the adhesive properties of sealers should however not overshadow the importance of their sealing properties [7]. The role of the root canal filling is to prevent the displacement of fluids, within or along the filling material, which could carry microorganisms and their by-products within the root canal towards the periapical tissues. The molecular structure and setting pattern of different sealers is broadly responsible for their dimensional behavior. The UDMA molecules are known to undergo significant polymerization shrinkage during setting [8]. Epoxy resins, on the contrary, demonstrate relatively good dimensional stability [9], and calcium silicate materials exhibit different degrees of porosity according to the characteristics of their setting environment [10].

The fluid transport method has been widely used for measuring the sealing efficacy of root fillings non-destructively and quantitatively [7, 11, 12]. The fluid transport model has been commonly operated with distilled water as a testing fluid [12, 13]. The introduction of a wetting fluid with a lower surface tension as testing fluid was a recent modification of the fluid transport method, which significantly improved its sensitivity [11].

The aim of the present study was to compare ex vivo, by means of a modified fluid transport model, the sealing efficacy of root fillings made by a single-cone technique with three different root canal sealers, after delayed post space preparation. As a control, a cold lateral compaction of gutta-percha with epoxy amine resin-based sealer was used.

## Materials and methods

### Sample preparation

One hundred extracted intact human mandibular premolars with a single root were selected. The teeth were extracted for reasons not related to this study, and the donors gave their permission that these teeth would be used for research purposes. The teeth were stored in water after extraction. Radiographs were exposed to confirm the presence of a single straight canal. Root canal diameters were measured radiographically in buccal-lingual and mesial-distal directions at 4 mm from the apex. According to the obtained dimensions, teeth were distributed by stratified randomization among the experimental groups 1, 2, 3, and 4 (*n* = 20/group). Twenty more teeth were selected for the negative (*n* = 10) and positive controls (*n* = 10). Kruskal-Wallis test (*p* > 0.05) confirmed the dimensional homogeneity among the groups.

The teeth were embedded in self-polymerizing methyl methacrylate resin cylinders (Dentimex, Zeist, the Netherlands) leaving the apical third of the root uncovered and were decoronated at 14 mm from the apex using an IsoMet 1000 saw (Buehler Ltd., IL, USA) under copious water cooling. The margins adjoining the root and the resin were sealed with cyanoacrylate glue (Permacol, Ede, the Netherlands). Except for the apical foramen and the coronal access, the entire specimen surface was covered with two layers of nail varnish. In the negative controls, the whole surface was covered.

### Root canal instrumentation

A size 15 K-file was inserted in the root canal until it was just visible at the apical foramen, and this length was recorded for each root. Working length was determined by subtracting 1 mm from this length and the preparation was conducted with a crown down technique using GT rotary instruments (Dentsply Maillefer, Ballaigues, Switzerland) to a size 40, 0.04 taper master file. At each change of file, canals were rinsed with 2 mL of 2 % sodium hypochlorite (NaOCl). After completion of the preparation, the canal was additionally rinsed with 2 mL of 2 % NaOCl, a size 15 ultrasonic K-file was inserted into the root canal 1 mm short of working length, and the irrigant was activated ultrasonically (P5-Suprasson; Satelec, Merignac, France) for 1 min at a power setting 4. The canals were then flushed with 0.5 mL 17 % EDTA solution, which was left in place for 1 min. Subsequently, the canals were rinsed with 2 mL of 2 % NaOCl followed by a final rinse with 2 mL of deionized water. The patency of the apical foramen was confirmed by inserting the tip of a size 15 K-file through it. All the experimental procedures were performed by the same endodontist (D. D. S.) to eliminate operator variables.

### Root filling

All root canal sealers were mixed and delivered according to the manufacturer’s recommendations. The tested sealers are listed in Table 1.

**Table 1:** Composition of the tested sealers

Sealer	Manufacturer	Delivery/mixture	Composition
AH26	Dentsply DeTrey, Konstanz, Germany	Powder/resin Manual	Bismuth oxide, methenamine, silver, titanium oxide, epoxy resin
EndoSequence BC Sealer	Brasseler, Savannah, GA, USA	Premixed paste	Zirconium oxide, calcium silicates, calcium phosphate monobasic, calcium hydroxide, filler, thickening agents
EndoREZ	Ultradent, South Jordan, UT, USA	Paste/paste automix	Urethane dimethacrylate (UDMA), triethylene glycol dimethacrylate (TEGDMA), dimethyl propiothetin (DMPT), triethylene glycol dimethacrylate, tolylimino diethanol, zinc oxide, barium sulfate, pigments

- Group 1: single tapered gutta-percha cone and AH26 (*n* = 20)

The canals were dried with size 40 paper points. The AH26 sealer was mixed on a glass slab. A lentulo spiral was swiped through the AH26 sealer once, inserted into the root canal slightly short of working length, and rotated at low speed. A size 40 taper 0.04 master gutta-percha cone (MTwo, VDW GmbH, Munich, Germany) was lightly coated with the AH26 sealer and inserted to working length. The coronal excess of gutta-percha was severed with a heat carrier (Touch’n Heat, SybronEndo Corporation, Orange, CA, USA).

- Group 2: single tapered gutta-percha cone and EndoSequence BC Sealer (*n* = 20)

The canals were dried with size 40 paper points. The intra canal tip was inserted until binding point, slightly withdrawn, and the sealer was injected by compressing the plunger of the syringe while slowly withdrawing the tip. A corresponding size 40 taper 0.04 gutta-percha master cone was lightly coated with BC Sealer and carefully inserted into the canal to working length. The excess part of the cone was severed with a heat carrier (SybronEndo, USA).

- Group 3: single tapered EndoREZ cone and EndoREZ sealer (*n* = 20)

The canals were moderately dried with size 40 paper points as recommended by the manufacturer. The EndoREZ sealer (Ultradent Products, South Jordan, UT, USA) was injected in a syringe (Skini Syringe, Ultradent, USA) and injected into the canal with a NaviTip® needle placed slightly short of working length. The sealer was injected by compressing the plunger of the syringe while slowly withdrawing the needle. A size 40 taper 0.04 master EndoREZ cone (Ultradent Products, South Jordan, UT, USA) was lightly coated with the EndoREZ sealer and inserted to working length. The EndoREZ sealer was then light cured for 40 s. The coronal excess of EndoREZ cone was severed with a heat carrier (SybronEndo, USA).

- Group 4: cold lateral compaction and AH26 (*n* = 20)

The canals were dried with size 40 paper points. The AH26 was mixed on a glass slab. A lentulo spiral was swiped through the AH26 sealer once, inserted into the root canal slightly short of working length, and rotated at low speed. An ISO 40 gutta-percha cone (Dentsply Maillefer, Ballaigues, Switzerland) was fitted at working length and used as master cone. Cold lateral compaction was performed using a size B nickel-titanium finger spreader (Dentsply Maillefer) placed up to 1 mm short of the working length with the master cone in place and ISO size 20 accessory cones (Dentsply Maillefer) lightly coated with sealer. This procedure was conducted until the tip of the spreader could not go further than 2 mm from the coronal access. The filling material was then severed at the root canal entrance with a heat carrier (SybronEndo, USA).

- Positive controls (*n* = 10): instrumented root canals were filled with a loosely fitting gutta-percha cone without any sealer.
- Negative controls (*n* = 10): instrumented root canals were filled in the same manner as in group 1. The whole apical and coronal surfaces were sealed with nail varnish.
  All the samples were kept in 100 % humidity at 37 °C for 10 days to allow all the materials to set completely. The coronal part of the root filling was then removed with a heat carrier (SybronEndo, USA), leaving only the 4 most apical mm of the root filling in place.

### Fluid transport testing

**Fig. 1:**
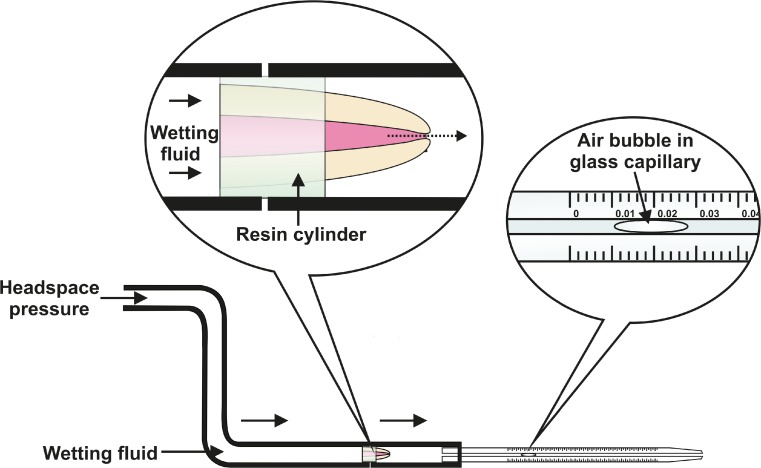
The schematic drawing of the fluid transport setup used in the present study

A modified fluid transport model as previously described was mounted (Fig. 1) [11]. A 20-kPa input pressure was applied to the coronal end of the root and Galpore (Benelux Scientific, Eke, Belgium) was used as testing fluid. Galpore is a perfluoroether with a very low surface tension (16 mN m^−1^) and a viscosity of 4.4 mPa.s. Fluid transport was measured by two of the researchers by observing the movement of an air bubble entrapped within a 0.1-mL glass capillary tube (Witeg Labortechnik GmbH, Wertheim, Germany). The fluid transport was recorded at room temperature at 30-min intervals for a total of 6 h. The mounting and measurements were performed in ventilated chambers, wearing gloves and goggles at all times.

### Statistical analysis

The fluid transport values did not follow a normal distribution, as determined by the Shapiro-Wilk test; therefore, non-parametric tests were used, and the results were provided as median with interquartile range. The overall comparisons between the different groups were made using a Kruskal-Wallis test and pairwise comparisons using a Mann-Whitney *U* test with Bonferroni correction. Chi-square test was conducted in order to compare the percentage of leaking specimens in each group. The level of significance was set at *p* < 0.05. Statistical analyses were performed using IBM SPSS Statistics version 21.0 (IBM Corp., Armonk, NY, USA).

## Results

No fluid transport could be measured in the negative control group whereas rapid fluid transport in the positive control group prevented recording but indicated massive leakage.

The median (interquartile range) in microliters per hour for fluid transport values are displayed in Table 2. The amount of fluid transport was significantly different between all groups (*p* < 0.001). No statistically significant difference between groups 1, 3, and 4 (*p* > 0.05) was detected. Group 2 demonstrated significantly more fluid transport than group 1 (*p* < 0.001), group 3 (*p* = 0.002), and group 4 (*p* = 0.023).

**Table 2:** Median and interquartile range for fluid transport values in microliter per hour and the percentage of specimens exhibiting fluid transport in each group with statistical comparisons

	Group 1 SC-AH26	Group 2 SC-BC	Group 3 SC-ER	Group 4 LC-AH26	*p* value
*n*	20	20	20	20	
% leaking samples	90^1^	95^1^	85^1^	35^2^	<0.001 (CS)
Median	0.25^a^	0.5^b^	0.17^a^	0^a^	<0.001 (KW)
Interquartile range	0.15–0.33	0.42–0.6	0.08–0.25	0–3.13	–

Sealers: AH26, BC (EndoSequence BC sealer), ER (EndoREZ). Different superscript numbers indicate significant differences. Different superscript letters indicate significant differences

*SC* single-cone, *LC* lateral compaction, *CS* chi-square, *KW* Kruskal-Wallis

The percentages of specimens demonstrating fluid transport were significantly different between the groups (*p* < 0.001) and are presented in Table 2. No statistically significant difference in percentage of specimens with fluid transport could be detected between the three single-cone groups whereas significantly fewer specimens exhibited fluid transport in the cold lateral compaction group.

## Discussion

Fluid transport is based on the penetration of fluid in through-and-through pores within or between the filling material and the dentinal wall in a root canal under light pressure [12]. Capillary action; entrapped air/liquid; and the fluid characteristics such as its pH, its molecular size, and its surface tension may affect the penetration of the fluid [11]. Surface tension is the force between molecules that produces a tendency for the surface area of a liquid to decrease, and it tends to inhibit the spread of a liquid over a surface and penetration ability of the liquid in a capillary tube. The use of liquids with lower surface tensions will result in an increase of the fluid penetration into the pores. According to Young’s equation, a pore can be seen as a cylindrical capillary of diameter *d*, and Pc being the Young’s capillary pressure of the entrapped air within the pore:

$$ Pc=\frac{4\sigma \cos \theta }{d} $$

This equation can be reformulated as $d=\frac{4\sigma \cos \theta }{Pc}$

At an applied pressure (Pc) of 20 kPa (20,000,000 mN m^−2^) and with a surface tension (*σ*) of 16 mN m^−1^ for the wetting fluid and an ‘assumed’ wetting angle of the wetting fluid with dentine (*θ*) of 0°, the narrowest pore diameter that could be detected is approximately 3.20 μm.

In the present study, fluid transport could be detected in a high proportion of the specimens. This could be explained not only by the use of a wetting liquid as testing fluid, which increases the sensitivity of the model [11], but also by the relatively limited length of the root filling. The placement of a post requires the removal of the coronal part of the root canal filling, which may decrease the sealing efficacy by reducing the length of the root filling and the area of the dentin-filling interface.

The sealing efficacy of EndoSequence BC Sealer was inferior to the other materials tested in this study. EndoSequence BC Sealer is a calcium silicate-based root canal filling material with alleged bioactive properties. A bioactive material is supposed to have the ability to provoke a specific biological response at the interface of the material with its surrounding, which would result in a stable bond [10]. Calcium silicate-based sealers need water to form calcium silicate hydrate and calcium hydroxide and to achieve setting [14]. While the presence of water in excess could create an unstable matrix resulting in high porosity, an insufficient amount of water could prevent complete setting [10, 15]. The calcium hydroxide formed by reaction with water is expected to react with the phosphate ions present in the environment and/or within the sealer to form a crystalline deposit of calcium phosphate on the material surface [6]. These deposits would be the base of a bond between the material and the dentin. The foundation of these reactions is the fluid present at the reaction sites. Thus, the type and amount of fluid available in the environment determine the rate and the end products of the reaction [10]. Variability in root canal anatomy, as well as heterogeneity of root canal dentin along the same root canal or among different teeth, makes moisture control inside the root canal highly unpredictable [16, 17]. Since dentin in the apical region of the root canal is usually sclerotic [16], the amount of moisture available from dentinal tubules in the apical third could be insufficient for calcium silicate-based sealers to achieve a proper setting. This may explain the difference between the present findings and a previous study that evaluated the sealing efficacy of a calcium silicate sealer in entirely filled roots [18]. In such a design and contrary to the present setup, a proper seal of the coronal part of the root may have masked the quality of the apical seal. Another study using scanning electron microscopy has demonstrated the formation of a tag-like structure on the surface of EndoSequence BC Sealer placed in 3-mm root sections immersed in phosphate-buffered saline (PBS) [6]. However, images selected at specific loci describe only those specific regions and can hardly represent the whole root canal, especially in terms of sealing efficacy. Furthermore, immersion of root sections instead of whole roots in PBS is quite an unrealistic simulation of the available moisture within the root canal.

EndoREZ is a methacrylate resin-based sealer with adhesive properties. Since it is hydrophilic and flowable, it can penetrate into the dentinal tubules [19]. The highly unfavorable cavity configuration of the root canal, however, results in excessive polymerization stresses within the material. This could lead to the formation of gaps, caused by the detachment of the sealer from dentin or from the obturation cone [20]. The distribution and dimensional patterns of these gaps are yet unknown. Their discontinuity along the root canal could provide an explanation for the similar performance of the methacrylate resin-based sealer specimens in comparison to the epoxy amine resin-based sealer specimens as evaluated by the fluid transport model.

Epoxy amine resin-based sealers such as AH26 are considered to possess excellent physical and chemical properties [21]. They present good dimensional stability and undergo limited polymerization shrinkage [9]. This could be attributed to their molecular structure as well as to their pattern of polymerization. The lower crosslink density in the epoxy molecules compared to the UDMA limits the number of crosslink network formation and thus polymerization shrinkage [22]. Also, epoxy molecules contain an oxirane ring. The ring opening during polymerization lowers shrinkage, while the delayed consumption of the reactive species could provide stress relaxation [23]. They are, therefore, often used as a gold standard to which other sealers could be compared.

Cold lateral compaction is an operator-dependent technique, often associated with unfilled spreader tracks [2]. These spreader tracks (voids) can serve as pathways of fluid transport and could explain the relatively high fluid transport values observed in few of the specimens in group 4. When the percentage of specimens with fluid transport was taken into account, there was no statistically significant difference between the number of specimens exhibiting fluid transport in the three single-cone groups, whereas significantly fewer specimens exhibited fluid transport in the cold lateral compaction group. Considering the similar (not significantly different) amount of fluid transport between the cold lateral compaction group and the single-cone group in combination with AH26, this is an interesting finding. One may speculate that through-and-through pores in the lateral compaction group were larger and more abundant in comparison to single-cone groups. The spreader tracks may be responsible for these larger pores. Furthermore, it is known that the forces generated during lateral compaction could also produce dentinal defects that could evolve into vertical root fracture [3]. In this sense, the lack of compaction component with the single-cone technique could reduce the risks of causing dentinal defects during the filling procedure.

A limitation of the present study is the fact that fluid transport was measured directly after the setting of the materials, and no longitudinal measurements were done. Since dimensional changes such as an increase or decrease in the diameters of the pores within and along the filling materials may occur over time, long-term measurements may provide different results [24].

## Conclusion

The sealing efficacy after delayed post space preparation of root fillings made by a single-cone technique in combination with the epoxy amine resin- or methacrylate resin-based sealers and that of root fillings made by a cold lateral compaction technique in combination with the epoxy amine resin-based sealer were similar and superior to that of root fillings made by a single-cone technique in combination with the calcium silicate-based sealer.

## References

[1] Schwartz RS, Robbins JW (2004). Post placement and restoration of endodontically treated teeth: a literature review. J Endod.

[2] Keles A, Alcin H, Kamalak A, Versiani MA (2014). Micro-CT evaluation of root filling quality in oval-shaped canals. Int Endod J.

[3] Shemesh H, Wesselink PR, Wu MK (2010). Incidence of dentinal defects after root canal filling procedures. Int Endod J.

[4] Barszcewska-Rybarek IM (2009). Structure-property relationships in dimethacrylate networks based on Bis-GMA, UDMA and TEGDMA. Dent Mater.

[5] Fisher MA, Berzins DW, Bahcall JK (2007). An in vitro comparison of bond strength of various obturation materials to root canal dentin using a push-out test design. J Endod.

[6] Han L, Okiji T (2013). Bioactivity evaluation of three calcium silicate-based endodontic materials. Int Endod J.

[7] Moinzadeh AT, Mirmohammadi H, Hensbergen IA, Wesselink PR, Shemesh H (2014). The correlation between fluid transport and push-out strength in root canals filled with a methacrylate-based filling material. Int Endod J.

[8] Hammad M, Qualtrough A, Silikas N (2008). Extended setting shrinkage behaviour of endodontic sealers. J Endod.

[9] Zhou HM, Shen Y, Zheng W, Li L, Zheng YF, Haapasalo M (2013). Physical properties of 5 root canal sealers. J Endod.

[10] Camilleri J, Grech L, Galea K, Keir D, Fenech M, Formosa L, Damidot D, Mallia B (2014). Porosity and root dentine to material interface assessment of calcium silicate-based root-end filling materials. Clin Oral Investig.

[11] Özok AR, Verhaagen B, Wesselink PR (2013). Improving the accuracy of a fluid transport method. Int Endod J.

[12] Monticelli F, Osorio R, Toledano M, Ferrari M, Pashley DH, Tay FR (2010). Sealing properties of one-step root-filling fibre post-obturators vs. two-step delayed fibre post-placement. J Dent.

[13] Wu MK, De Gee AJ, Wesselink PR (1994). Fluid transport and dye penetration along root canal fillings. Int Endod J.

[14] Camilleri J (2011). Characterization and hydration kinetics of tricalcium silicate cement for use as a dental biomaterial. Dent Mater.

[15] Loushine BA, Bryan TE, Looney SW, Gillen BM, Loushine RJ, Weller RN, Pashley DH, Tay FR (2011). Setting properties and cytotoxicity of a premixed bioceramic root canal sealer. J Endod.

[16] Özok AR, Wu MK, Wesselink PR (2002). Comparison of the in vitro permeability of human dentine according to the dentinal region and the composition of the simulated dentinal fluid. J Dent.

[17] Mjör IA, Smith MR, Ferrari M, Mannocci F (2001). The structure of dentine in the apical region of human teeth. Int Endod J.

[18] Ersahan S, Aydin C (2013). Solubility and apical sealing characteristics of a new calcium silicate-based root canal sealer in comparison to calcium hydroxide-, methacrylate resin- and epoxy resin-based sealers. Acta Odontol Scand.

[19] Tay FR, Loushine RJ, Monticelli F, Weller RN, Breschi L, Ferrari M, Pashley DH (2005). Effectiveness of resin-coated gutta-percha cones and a dual-cured, hydrophilic methacrylate resin-based sealer in obturating root canals. J Endod.

[20] Tay FR, Loushine RJ, Lambrechts P, Weller RN, Pashley DH (2005). Geometric factors affecting dentin bonding in root canals: a theoretical modelling approach. J Endod.

[21] Baldi JV, Bernardes RA, Duarte MA, Ordinola-Zapata R, Cavenago BC, Moraes JC, de Moraes IG (2012). Variability of physicochemical properties of an epoxy resin sealer taken from different parts of the same tube. Int Endod J.

[22] Kurdikar DL, Peppas NA (1995). The volume shrinkage, thermal and sorption behaviour of polydiacrylates. Polymer.

[23] Guggenberger R, Weinmann W (2000). Exploring beyond methacrylates. Am J Dent.

[24] De Bruyne MAJ, De Bruyne RJ, De Moor RJ (2006). Long-term assessment of the seal provided by root-end filling materials in large cavities through capillary flow porometry. Int Endod J.

